# An Energy-Efficient LiDAR Receiver Using Time-to-Voltage Converter and SAR ADC in 180 nm CMOS

**DOI:** 10.3390/mi17050622

**Published:** 2026-05-19

**Authors:** Bobin Seo, Sung-Min Park

**Affiliations:** Division of Electronic & Semiconductor Engineering, Ewha Womans University, Seoul 03760, Republic of Korea; qhqls28@ewha.ac.kr

**Keywords:** TIA, time-to-voltage converter, LiDAR, SAR ADC, CMOS

## Abstract

This paper proposes an energy-efficient LiDAR receiver topology based on a time-to-voltage converter (TVC) followed by a 5-bit SAR ADC. By converting the time-interval between START and STOP signals into the voltage domain, the proposed topology avoids the complexity of conventional TDC-based designs and enables the use of a moderate-speed, energy-efficient SAR ADC. The proposed TVC in the proposed LiDAR receiver consists of an on-chip avalanche photodiode (APD), a CMOS transimpedance-limiting amplifier (CTLA), a time-gating circuit, a ramp generator, and a peak-and-hold (PDH) block. Thereafter, the converted voltages are digitized by a VCM-based single-ended SAR ADC with a binary-weighted CDAC, a strong-arm latch comparator, and custom digital logic. A reset generator is also incorporated to coordinate the sampling, comparison, and settling phases. The proposed LiDAR receiver is implemented in a 180 nm CMOS process, where the TVC occupies an area of 171 μm × 98.8 μm, while the TVC-SAR receiver occupies 417 μm × 356 μm, respectively. The proposed LiDAR receiver consumes 13 mW from a single 1.8 V supply, in which the SAR ADC consumes 3.68 mW only. The TVC-SAR receiver resolves the time-intervals ranging from 7 ns to 32.1 ns with a resolution of 0.81 ns. Hence, the proposed topology provides an energy-efficient solution along with its reduced circuit complexity and chip implementation for short-range LiDAR applications.

## 1. Introduction

Light detection and ranging (LiDAR) technology has become increasingly important in a wide range of applications, including transportation and autonomous systems, environmental and geospatial monitoring, satellite-based atmospheric observation, and healthcare monitoring such as activity recognition and fall detection [1,2,3,4,5,6]. These applications require highly accurate, fast, low-power, and cost-effective sensing solutions, rendering LiDAR sensors an essential component in modern electronic systems. As the adoption of LiDAR continues to expand, there has been a growing demand for compact and energy-efficient circuit implementations that can maintain reliable performance under strict power and size constraints.

In time-of-flight (ToF) LiDAR sensor systems, CMOS implementation often requires substantial downstream digital processing for timing extraction and digitization [7]. In some conventional architectures, these functions are performed using either on-chip or off-chip processing units. To address this issue, time-to-digital converters (TDCs) have been widely adopted in recent LiDAR systems [8,9,10]. However, TDC-based implementations usually involve considerable design complexity, and moreover their timing accuracy can be sensitive to mismatch, process-voltage-temperature (PVT) variations, metastability, and layout/routing parasitic components [11,12].

To alleviate these limitations, this paper proposes a topology in which a successive-approximation-register analog-digital-converter (SAR ADC) is placed after a time-to-voltage converter (TVC). The SAR ADC is an attractive choice because of its simple architecture and high energy-efficiency, particularly in low-to-medium-speed applications because the TVC first converts the time-interval between START and STOP signals into a voltage-domain quantity.

**Figure 1:**
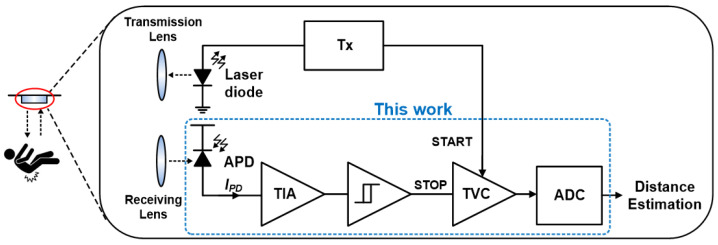
Block diagram of the proposed LiDAR sensor.

Figure 1 shows the block diagram of the proposed LiDAR receiver, where the SAR ADC is connected to the output of the TVC so that the time-intervals can be directly translated into digital codes.

**Figure 2:**
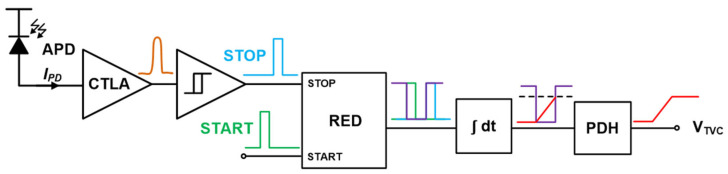
Block diagram of the TVC circuit.

Figure 2 shows the block diagram of the proposed TVC circuit, which consists of an on-chip avalanche photodiode (APD), a CMOS transimpedance-limiting amplifier (CTLA), a Schmitt trigger, a time-gating circuit called rising edge detector (RED), an enable-controlled ramp generator (i.e., an integrator circuit), and a peak-and-hold (PDH) block. Therefore, the TVC converts the time-intervals between the START signals transmitted from the transmitter (Tx) and the STOP signals reflected from the targets into the corresponding voltages, hence enabling the voltage-domain time-encoding for ToF LiDAR sensor applications [13]. Thereafter, a common-mode voltage (VCM)-based SAR ADC is employed as a back-end digitizer.

This paper is organized as follows. Section 2 discusses the circuit operations and, Section 3 presents the post-layout simulation results, and the conclusion is followed in Section 4.

## 2. Circuit Description

### 2.1. Front-End Detection

In this work, on-chip APDs are implemented based on a P^+^/N-Well/Deep N-Well (P^+^/NW/DNW) structure where avalanche multiplication is initiated at the P^+^/NW junction. The central P^+^ contacts are tied to the TIA input, suppressing slow substrate diffusion currents while maintaining high responsivity and wide bandwidth. Shallow-trench-isolation (STI) formed deeper than the junction serves as a guard ring, thus reducing electric field crowding and preventing premature breakdown at the cost of slightly worse responsivity. The DNW enhances the NIR sensitivity by blocking hole diffusion into the P-substrate via a built-in potential barrier. For optical access, salicide blocking is applied to the P^+^ source/drain regions, while P^+^ contacts remain silicided for low resistance. Minimizing the contact area is also necessary to preserve responsivity [14,15].

**Figure 3:**
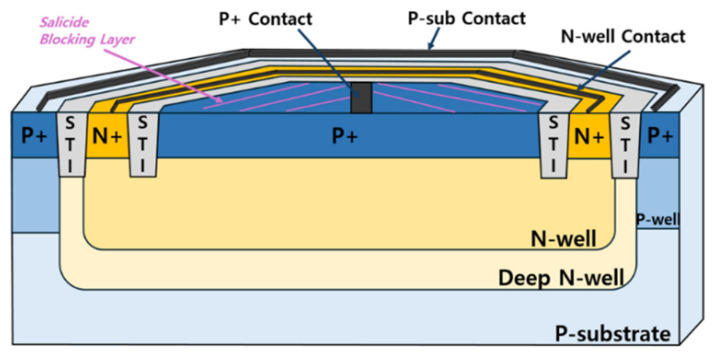
Internal cross-section of the P^+^/NW/DNW APD [14,15].

Figure 3 shows the internal cross-section of the proposed P^+^/NW/DNW APD. For improved optical and electrical performance, salicide blocking is applied to the P^+^ source/drain regions to enable efficient light absorption, while the P+ contacts remain silicided to reduce series resistance. The active area diameter is designed to be 40 μm to enhance responsivity, and an octagonal layout is adopted to mitigate edge breakdown. The device achieves a responsivity of 4.16 A/W at a reverse bias of 10.5 V and a photodetection bandwidth of 1.7 GHz at 10.25 V.

**Figure 4:**
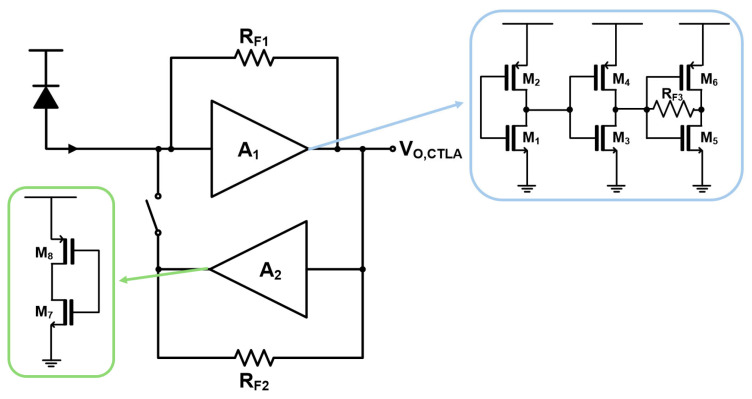
Schematic diagram of the CTLA [16,17].

Figure 4 illustrates the schematic diagram of the CMOS transimpedance-limiting amplifier (CTLA) employed as the front-end amplifier. The CTLA adopts a dual-feedback topology composed of a main inverter-based TIA and an additional active feedback path. An NMOS switch is inserted in the feedback loop so that the auxiliary feedback is activated only when the input photocurrent exceeds a certain threshold [16,17]. Under small-signal input conditions, the circuit operates as a conventional TIA to maintain linear amplification, while for large input currents the active feedback path drives the output into saturation, thus performing a limiting function. This approach suppresses pulse distortion and extends the input dynamic range without requiring a multi-stage limiting amplifier. The CTLA exhibits a transimpedance gain of 69.8 dBΩ and a bandwidth of 2.78 GHz, while consuming 6.76 mW. The input-referred noise spectral density is 14.9 pA/√Hz, and the input dynamic range is 40 dB (10 µA_pp_ to 1 mA_pp_).

**Figure 5:**
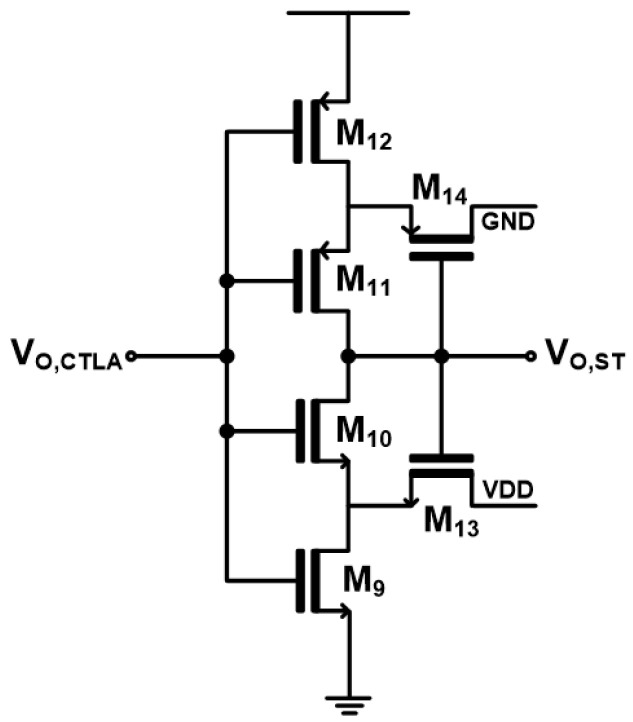
Schematic diagram of the Schmitt trigger.

Figure 5 shows the schematic diagram of the Schmitt trigger that is employed in the proposed TVC to convert the analog output of the CTLA into a digital signal. Compared to a conventional latch-based design, the suggested Schmitt trigger can be realized using a smaller number of CMOS transistors, enabling a more compact and power-efficient design. In this work, the circuit uses only six CMOS transistors.

Due to its inherent hysteresis characteristics, the Schmitt trigger improves noise immunity and prevents false switching caused by small fluctuations in the TIA output. This effectively allows reliable signal detection over a wider input range, which can be interpreted as an improvement in the usable dynamic range of the TIA output. Although the Schmitt trigger consumes 1.02 mW, which is not particularly low for a simple six-transistor implementation, it provides a simpler and more power-efficient solution when compared to conventional approaches in time-based conversion systems that rely on more complex latch or comparator structures for analog-to-digital pulse generation.

### 2.2. Time-to-Voltage Converter

**Figure 6:**
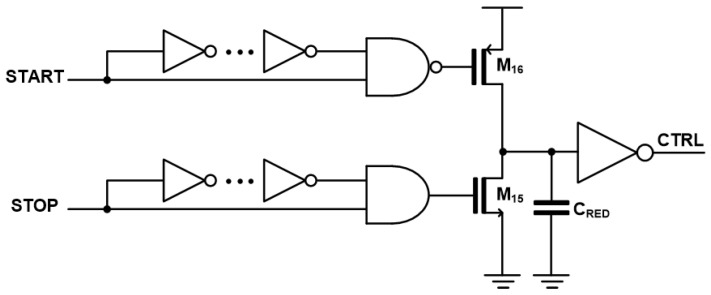
Schematic diagram of the time-gating circuit.

Figure 6 illustrates the schematic diagram of the time-gating circuit, where START and STOP signals pass through inverter stages to introduce a slight delay, thereby generating narrow pulse signals based on edge-detection principles. Unlike a conventional rising-edge detector that only indicates the occurrence of a signal transition, the proposed circuit utilizes both START and STOP signals to capture the time-interval between them.

In this implementation, the STOP signal corresponds to V_O,ST_, while the START signal is externally applied from the Tx. These signals drive the gates of the PMOS and NMOS transistors, which selectively connect the capacitor node to VDD or GND. Consequently, the capacitor is charged during the time-interval between START and STOP, effectively converting the time-difference into a voltage level. The inverted voltage across the capacitor is then used as the CTRL signal.

**Figure 7:**
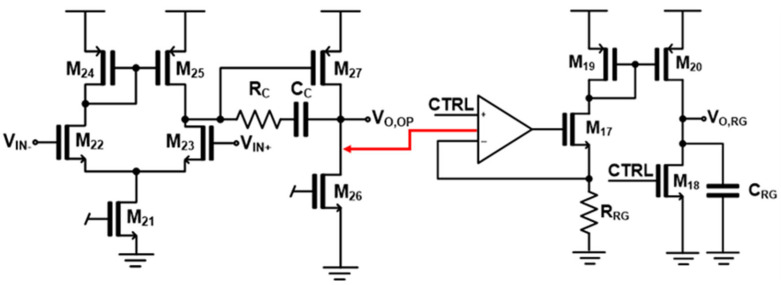
Schematic diagram of the time-gated ramp generator circuit.

Figure 7 shows the schematic of the proposed ramp generator that operates as a time-gated integrator rather than a conventional continuous-time ramp generator. It consists of a voltage-to-current conversion stage (op-amp, a resistor, and M_17_), a current mirror (M_19_–M_20_), a load capacitor (C_RG_) and a reset switch (M_18_). The op-amp regulates the source node of M_17_ such that a current proportional to the input voltage is continuously generated through M_17_. This current is mirrored by M_19_–M_20_ and serves as the charging current for the load capacitor (C_RG_). The CTRL signal generated from the time-gating circuit defines the integration window and operates as an active-low enable signal.

When CTRL is high, M_18_ is turned-on, thus forcing the output node to ground and resetting the capacitor. In this phase, the mirrored current is diverted through M_18_, and no integration occurs.

When CTRL transitions to low (i.e., during the time-interval between START and STOP signals), M_18_ is turned-off and the mirrored current from M_20_ is injected into the capacitor. In consequence, the capacitor is actively charged during this interval, generating a voltage ramp proportional to the input time difference. Therefore, the proposed circuit can generate ramp signals only within the defined time-interval, hence enabling accurate time-to-voltage conversion for the subsequent SAR ADC.

**Figure 8:**
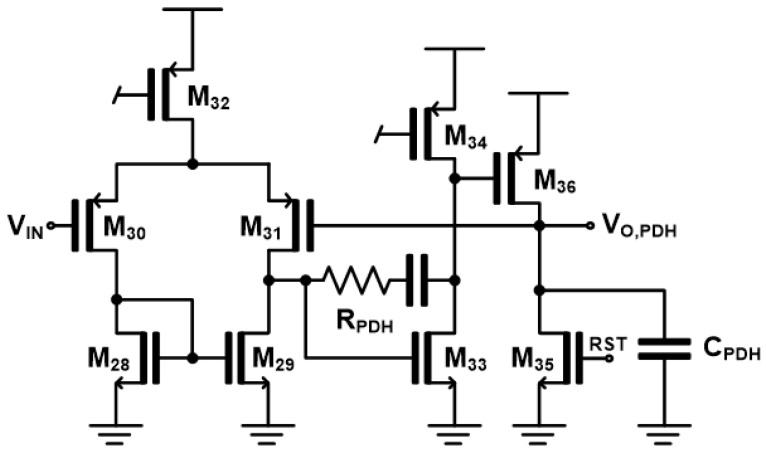
Schematic diagram of the peak-detect and hold (PDH) circuit.

Figure 8 shows the peak detect-and-hold (PDH) circuit where the input of the PDH (V_IN_) is connected to V_O,RG_, so that the circuit detects and holds the peak voltage stored on the capacitor. The reset signal (RST) discharges the stored voltage on the capacitor and is externally applied after two cycles of the SAR ADC to ensure stable operations. This configuration enables accurate sampling of the peak value and maintains it for subsequent processing by the ADC, thereby preventing signal loss due to rapid signal variations.

**Figure 9:**
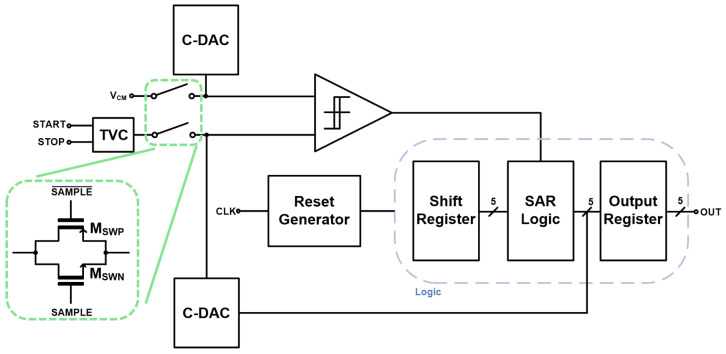
Block diagrams of the proposed TVC-SAR receiver.

Figure 9 illustrates the block diagram of the proposed TVC-SAR receiver. As aforementioned, START signals from the Tx are applied directly to the TVC whereas STOP pulses reflected from targets enter the TVC after being processed through the analog front-end circuit including the on-chip APD and the CTLA. The TVC converts the time-intervals between the START and STOP signals into analog voltages. Then, the generated voltages are transferred to the SAR ADC through a sample-and-hold (S/H) circuit followed by a VCM-based single-ended SAR ADC.

The SAR ADC consists of an S/H circuit that samples both IN+ and IN− inputs (which correspond to the comparator’s differential inputs), a capacitive digital-to-analog converter (C-DAC), a strong-arm latch comparator, a reset generator, and digital logic. To maintain sampling consistency, the VCM is sampled by using a C-DAC. The S/H circuit is implemented using a transmission-gate (TG) based sampling structure, and is controlled by the ‘Sample’ and ‘Sample_B’ signals generated from the logic block of the SAR ADC.

### 2.3. Strong-Arm Comparator and SR Latch

**Figure 10:**
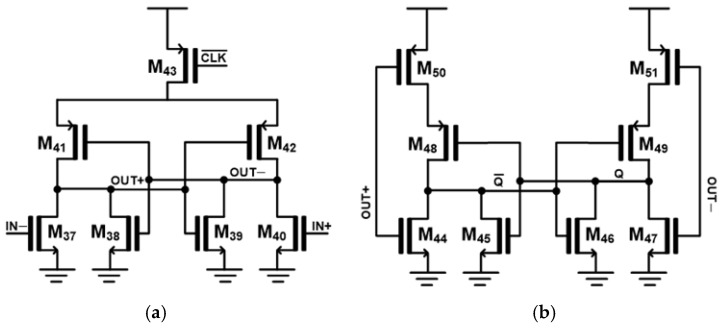
Schematic diagrams of (**a**) strong-arm comparator and (**b**) SR latch.

Figure 10a depicts the schematic diagram of the strong-arm comparator. Whenever IN+ is greater than IN−, the transistor M_40_ is turned on. Therefore, a low signal is delivered to both M_38_ and M_41_, turning M_38_ off and M_41_ on. In consequence, the output node (OUT+) is pulled up to V_DD_, producing a strong logic high signal. To the contrary, the opposite output node (OUT-) is discharged toward ground, generating a strong logic low signal. These regenerative operations rapidly amplify the small input difference and enable fast decision-making. In addition, the clock signal (CLK) is applied to the transistor M_15_, which acts as a tail switch. The comparator operates only when the clock signal is high, thereby reducing static power consumption and improving overall power efficiency.

Figure 10b shows the schematic diagram of the SR latch. By placing the SR latch after the strong-arm comparator, the comparison result is stored and maintained until the next clock cycle, ensuring stable digital output for subsequent logic processing. The SR latch is implemented by using NOR gates constructed at the MOSFET level, which improves the circuit uniformity and allows more consistent device matching when compared to gate-level implementations. Furthermore, the SR latch prevents the output glitches and provides a reliable digital interface between the analog comparator and the following digital logic.

In this work, the strong-arm latch is driven by $\bar{CLK}$, i.e., the inverted clock signal, instead of the main clock used in the preceding logic blocks. By operating on the complementary clock phase, the latch functions at a different timing from the logic that generates signals synchronized with the main clock signal. This timing separation provides additional settling time for the comparator outputs and helps to prevent potential timing errors, thereby ensuring more reliable and accurate comparison results.

### 2.4. C-DAC

As aforementioned, a V_CM_-based single-ended SAR ADC architecture is employed in the proposed TVC-SAR receiver. For proper V_CM_-based operations, the negative input (IN−) node is connected to the common-mode voltage (V_CM_), while the positive input (IN+) node is connected to V_in_ that is the output of the preceding TVC. The IN-node in this C-DAC is solely connected for symmetry and therefore does not affect the switching operations, comparator decision, or conversion, hence serving only to sample and hold the common-mode voltage (V_CM_).

**Figure 11:**
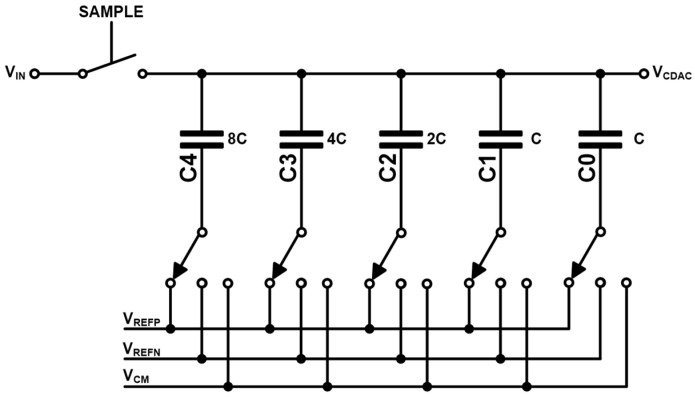
Schematic diagram of the C-DAC circuit.

Figure 11 shows the schematic diagram of the C-DAC. Here, the unit capacitance (C) has a minimum value of 35.6 fF. During the sampling phase, the top plate of the C-DAC is connected to V_in_ while the bottom plate is tied to V_CM_, thus allowing the input signal (V_in_) to be stored. Then, the input signal is sampled at the top plate of the C-DAC through the connected sample-and-hold (S/H) circuit. According to the bit decision generated by the SAR logic, the bottom plate of the C-DAC is switched to either V_REFP_ or V_REFN_.

After the sampling phase, the SAR conversion process begins. Starting from the most significant bit (MSB), the comparator sequentially performs bit-by-bit comparisons. The resulting C-DAC voltage (V_CDAC_) is compared with the input signal (V_in_) through the comparator to determine each bit. The total capacitance of the C-DAC array is designed to be 16C that corresponds to the binary-weighted capacitor structure, i.e., (8C + 4C + 2C + C + C). In the MSB decision step, the C-DAC output is adjusted by ±(V_REFP_ − V_REFN_)/2, depending on the switching result. In the subsequent steps, the voltage change becomes ±(V_REFP_ − V_REFN_)/4, ±(V_REFP_ − V_REFN_)/8, and so on for the lower bits. It is noted that the value of V_CM_ is defined as the mid-point between V_REFP_ and V_REFN_.

**Figure 12:**
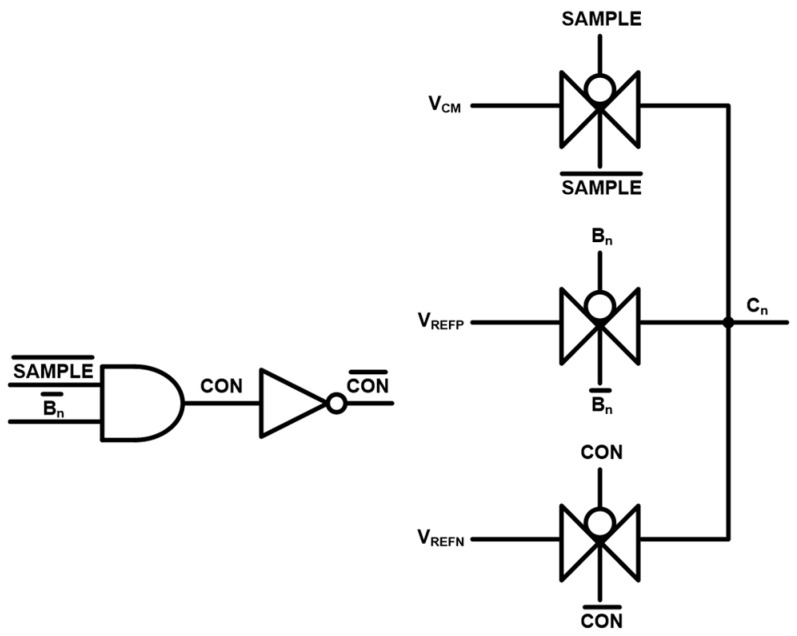
Schematic diagram of the switching control circuit of C-DAC.

Figure 12 depicts the switching control circuit for the C-DAC. Through this switching block, each capacitor is connected to V_CM_ during the sampling phase, allowing the input signal to be properly stored. During the conversion phase, based on the bit-decision result, the capacitor is switched to V_REFP_ when the decision becomes ‘1’ and to V_REFN_ when the decision becomes ‘0’. These switching operations enable the C-DAC to generate the corresponding reference voltage levels for each bit, hence facilitating the accurate successive approximation during the conversion process.

**Figure 13:**
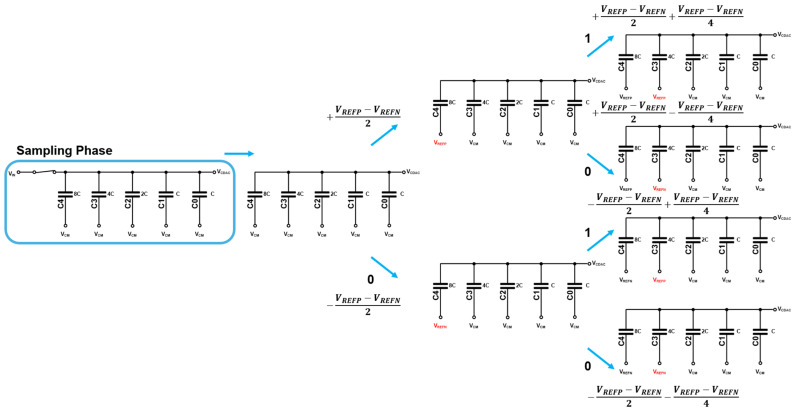
Schematic diagram of the C-DAC operations during SAR conversion.

Figure 13 illustrates the principal operations of the C-DAC for the upper 2-bit example. During the sampling phase, V_in_ is sampled onto the capacitors. Afterward, the input signal is disconnected, and the comparator determines the polarity between V_IN+_ and V_IN−_. Based on the decision, the capacitor is switched from V_CM_ to either V_REFP_ or V_REFN_, resulting in a step change of ±(V_REFP_ − V_REFN_)/2 in the C-DAC output for the MSB decision. In the subsequent bit cycle, the C-DAC output is further adjusted by ±(V_REFP_ − V_REFN_)/4.

### 2.5. Digital Logic and Reset Generator

**Figure 14:**
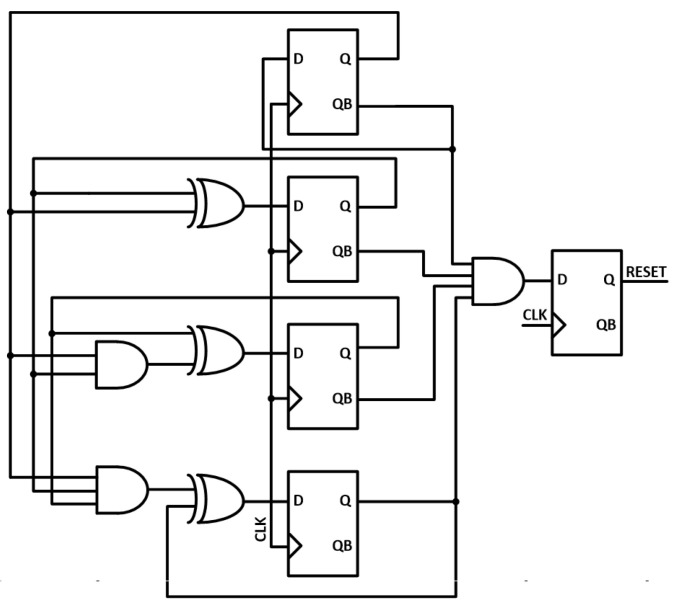
Schematic diagram of the reset generator.

Figure 14 depicts the block diagram of the reset generator circuit that consists of a D flip-flop (DFF) and logic gates. It can count eight clock cycles and generate a reset signal at the 9th cycle. In the proposed SAR ADC architecture, the sampling phase occupies two clock cycles, followed by five comparison cycles. Thereafter, one additional clock cycle is allocated for settling before the end-of-conversion (EOC) signal is generated. At every 9th rising edge of the clock, the reset signal is generated, returning the SAR ADC to the sampling phase. Although the proposed converter is a 5-bit SAR ADC, the reset signal is generated every eight clock cycles rather than six cycles in order to allocate additional timing for the end-of-conversion (EOC) signal generation and the SAMPLE state control. The generated reset signal is distributed to the digital logic block, where it initializes the internal registers and coordinates the timing of the SAMPLE and EOC signals.

**Figure 15:**
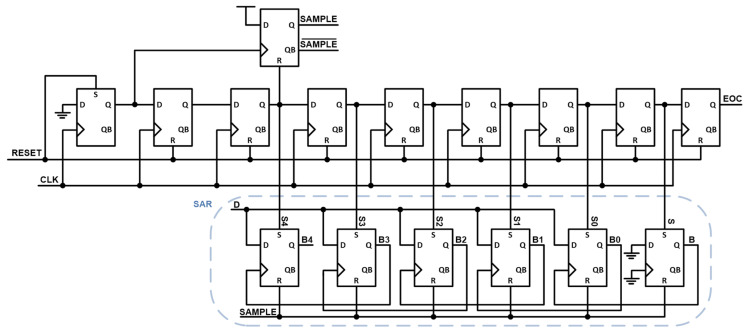
Block diagram of the digital logic.

Figure 15 shows the block diagram of the digital logic, consisting of a shift register and several DFFs that perform the bit-decision operation of the SAR algorithm. To ensure sufficient sampling and settling time before the sampled signal is transferred from the sample-and-hold (S/H) circuit to the C-DAC, the SAMPLE signal occupies twice the duration of a single bit-comparison state. Consequently, the sampling operation lasts for two clock cycles, followed by five clock cycles dedicated to the bit-by-bit comparison process. After the final comparison, an additional clock cycle is consumed to allow for redundancy in the bit decision and sufficient settling time. Although this results in more than five clock cycles being used for the conversion, the removal of redundancy improves the overall accuracy. Moreover, since the target ADC is not designed for high-speed operations, the additional clock cycle does not significantly degrade performance.

During the conversion phase, the SAR logic receives the comparator output and determines the switching direction of the C-DAC. Depending on whether the comparator output is logic ‘1’ or ‘0’, the corresponding capacitor is connected to either V_REFP_ or V_REFN_, respectively. In addition, the set input of each DFF is controlled by the output of the shift register so that the decision-making process is activated only for the corresponding bit position, while the other stages remain inactive. This mechanism enables the sequential bit evaluation and the proper propagation of the SAR decision through the register chain.

## 3. Layout and Simulation Results

**Figure 16:**
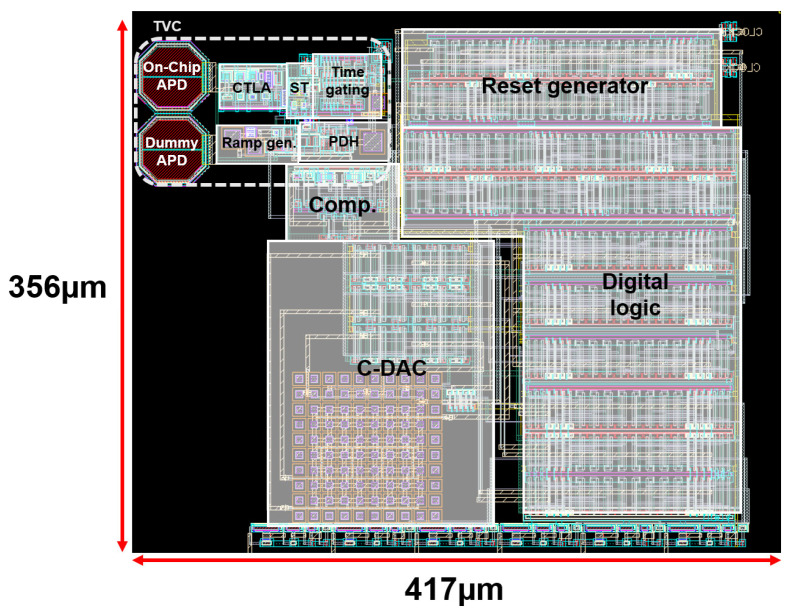
Layout of the proposed TVC-SAR receiver.

Figure 16 illustrates the layout of the proposed TVC-SAR receiver, in which the TVC block integrated with two on-chip P^+^/NW/DNW APDs occupies an area of 171 μm × 98.8 μm, while the overall TVC-SAR receiver layout occupies an area of 417 μm × 356 μm, respectively. It should be noted that a dummy APD is added not only to enhance the responsivity of the on-chip APD, but also to improve the symmetry of the analog front-end circuitry. Also, it is clearly seen that the layout of the proposed TVC-SAR receiver includes a TVC followed by a 5-bit SAR ADC. Post-layout simulations were carried out for the TVC-SAR receiver by using the model parameters based on a standard 180 nm CMOS technology. The DC simulation results indicate that the proposed TVC-SAR receiver consumes 13 mW from a single 1.8 V supply voltage, in which the SAR ADC only consumes 3.68 mW.

**Figure 17:**
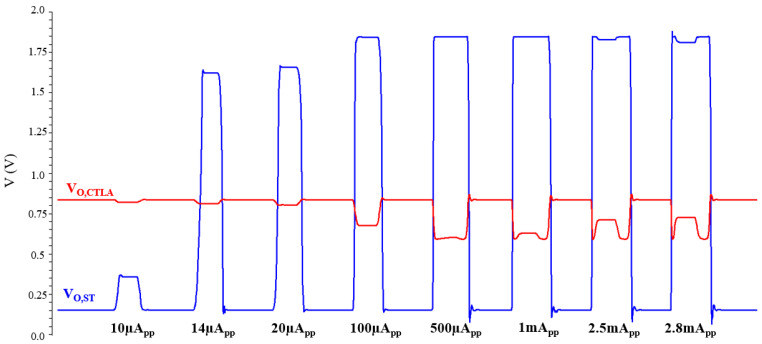
Pulse response comparison of the CTLA alone with the CTLA plus Schmitt trigger for various input currents.

Figure 17 compares the simulated pulse response of the CTLA alone with that of the CTLA plus Schmitt trigger for various input currents. As observed from the results, the Schmitt trigger starts to recognize the input signals from ~14 µA_pp_. This corresponds to a time-interval of 32.9 ns, which translates to a distance of 4.93 m. In the case of the CTLA alone, the output pulse begins to exhibit distortion starting from around 1 mA_pp_. However, with the addition of the Schmitt trigger, the distorted pulses are effectively restored, maintaining a stable pulse shape up to ~2.8 mA_pp_, confirming enhancement in the effective dynamic range. At 2.8 mA_pp_, the corresponding time-interval is 2.32 ns, which translates to a distance of 0.35 m.

**Figure 18:**
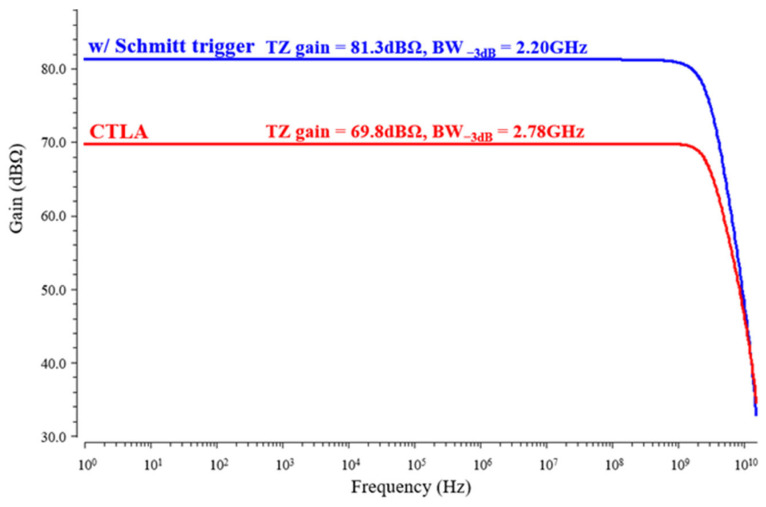
AC response comparison of the CTLA alone and the CTLA plus Schmitt trigger.

Figure 18 presents the simulated AC responses of the CTLA alone and the CTLA + Schmitt trigger, respectively. The inclusion of the Schmitt trigger increases the effective transition gain up to 81.3 dBΩ. While the Schmitt trigger is inherently non-linear, this AC response characterizes its small-signal behavior during switching, thus confirming that the system maintains a high bandwidth of 2.2 GHz for high-speed pulse restoration.

**Figure 19:**
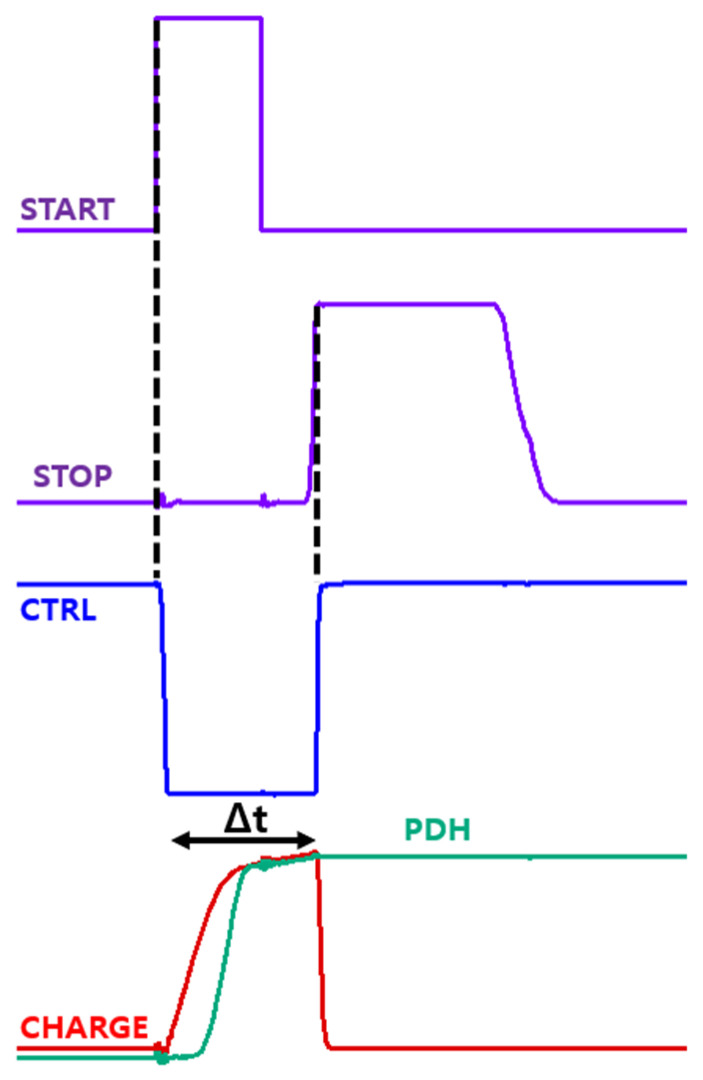
Simulation results of the proposed TVC for Δt = 7 ns.

Figure 19 shows the simulation results of the proposed time-to-voltage converter (TVC) with Δt = 7 ns. The START and STOP signals are detected at their rising edges to generate the CTRL signal, which remains low only during the interval Δt and stays high otherwise. This CTRL signal drives the ramp generator-based integrator, enabling charging exclusively during Δt. The resulting V_O,RG_ is then processed by the peak-detect-and-hold (PDH) circuit, effectively converting the input time interval into a proportional voltage.

**Figure 20:**
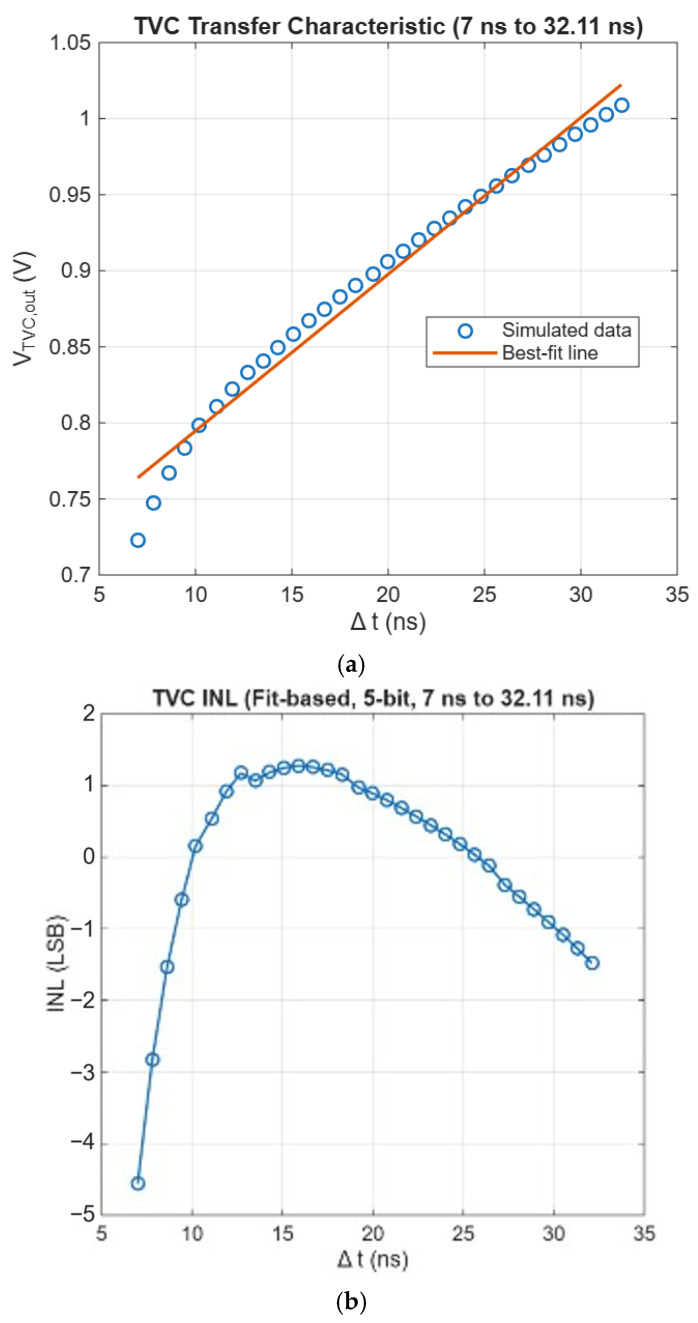

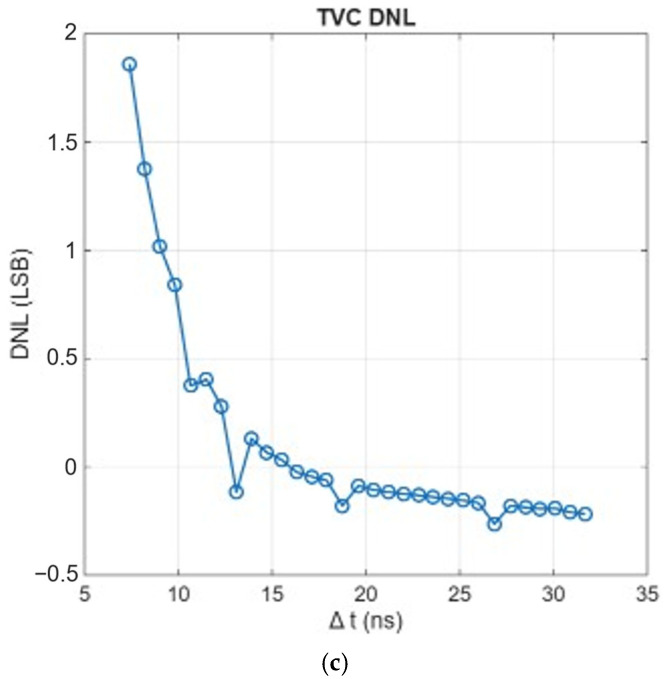
Simulation (**a**) transfer characteristic vs. best-fit line, (**b**) INL, and (**c**) DNL results of the proposed TVC.

Figure 20a depicts the transfer characteristic from the input time-interval (Δt) to the TVC output voltage, along with its best-fit linear approximation. While the overall trend follows a linear relationship, noticeable deviation is observed particularly in the lower time region due to the intrinsic nonlinear behavior of the TVC.

Figure 20b reveals the corresponding integral nonlinearity (INL), calculated with respect to the best-fit line and normalized by the 5-bit equivalent LSB. Over the evaluated range, the INL is simulated to be +1.276/−4.557 LSB, resulting in a peak-to-peak INL of 5.833 LSB. The relatively large INL mainly originates from the nonlinear voltage-time characteristic in the initial charging region.

To further evaluate the local linearity, the differential nonlinearity (DNL) was extracted based on the variation in the instantaneous slope relative to the ideal slope obtained from linear fitting.

Figure 20c shows the simulated results of DNL, where the DNL ranges from −0.264 to +1.860 LSB, with a peak-to-peak value of 2.124 LSB. Despite the non-ideal linearity reflected in the INL, the TVC maintains a monotonic transfer characteristic across the entire operating range, without any missing codes. This is also supported by the bounded DNL behavior, indicating that the local step variation remains controlled. Therefore, although the absolute linearity is limited, the proposed TVC is still suitable for applications where monotonicity and consistent time resolution are more critical than strict linearity.

**Figure 21:**
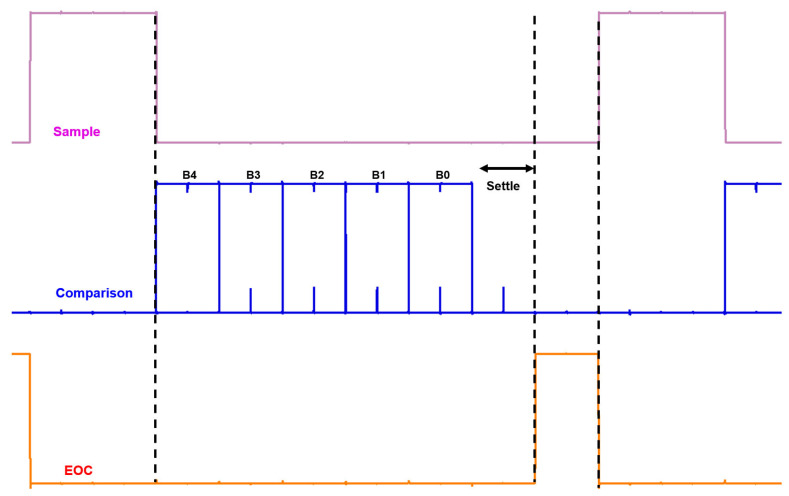
Simulated timing diagrams of the proposed SAR ADC operation phases.

Figure 21 presents the simulated phase operations of the proposed SAR ADC. The operation consists of a 2-clock-cycle sampling phase, followed by 5 comparison cycles. Afterward, one clock cycle is allocated for settling, during which the EOC signal is generated. This sequence repeats periodically. In the proposed implementation, the SAR ADC operates with a fixed synchronous timing scheme without an explicit asynchronous start-of-conversion (SOC) signal, while the PDH circuit holds the converted voltage before the SAR conversion phase begins.

**Figure 22:**
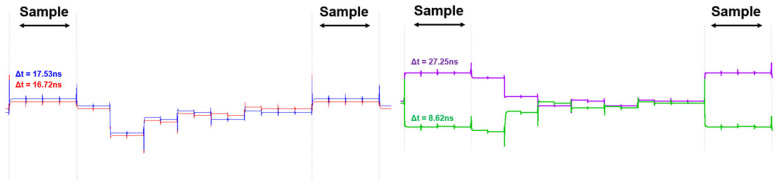
Simulated V_CDAC_ for different time intervals, respectively.

Figure 22 shows the simulated C-DAC output (V_CDAC_) for different time intervals. The results correspond to Δt = 8.62 ns, 16.72 ns, 17.53 ns, and 27.25 ns. The first sample (blue and red) on the left presents the results for 16.72 ns and 17.53 ns. It demonstrates that the SAR ADC can clearly distinguish even a small time-difference of 0.81 ns that corresponds to the LSB resolution. This LSB resolution of 0.81 ns corresponds to the detection range of 12.2 centimeters in distance. Therefore, it is noted that the proposed sensor system has a minimum detectable range of 1.05 m and a maximum detectable range of 4.82 m. The second sample (purple and green) on the right figure shows the results for 8.62 ns and 27.25 ns, where different switching behaviors of the C-DAC can be observed depending on the input time-interval.

**Figure 23:**
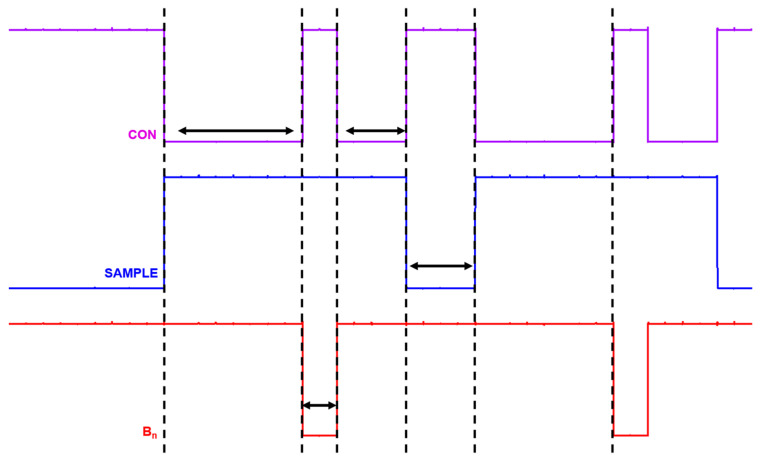
Timing diagrams of the C-DAC switching control signals.

Figure 23 shows the timing-diagrams of the C-DAC switching control circuit. The signals include B_n_ (generated by the SAR logic based on bit decisions), which switches the capacitor to V_REFP_, CON (that is connected to V_REFN_), and SAMPLE (that is connected to V_CM_). These timing-diagrams confirm that the capacitor switching occurs without overlap, thus ensuring proper and reliable C-DAC operations.

**Figure 24:**
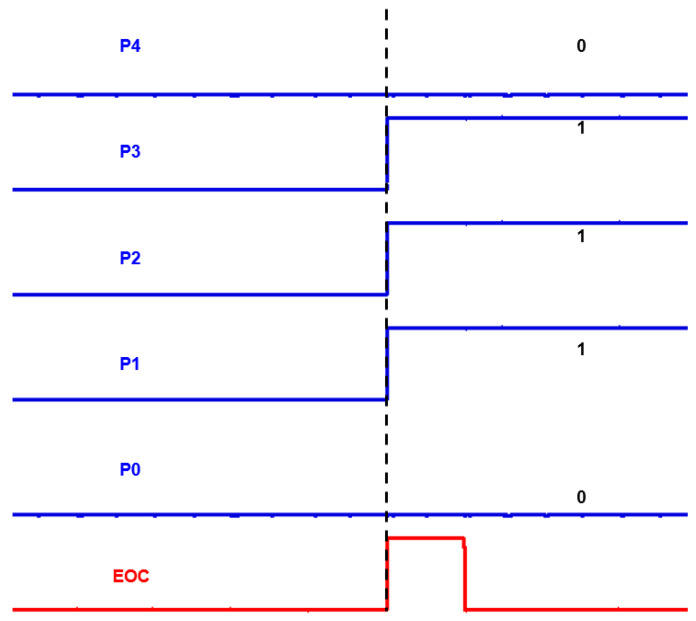
5-bit SAR ADC output waveforms at Δt = 18.34 ns.

Figure 24 presents an example of the full 5-bit digital output for Δt = 18.34 ns. It can be observed that the final output is determined and becomes valid simultaneously with the assertion of the end-of-conversion (EOC) signal. In addition, all output bits (P_n_) exhibit clearly distinguishable logic low and high levels, indicating stable and accurate digital conversion.

**Figure 25:**
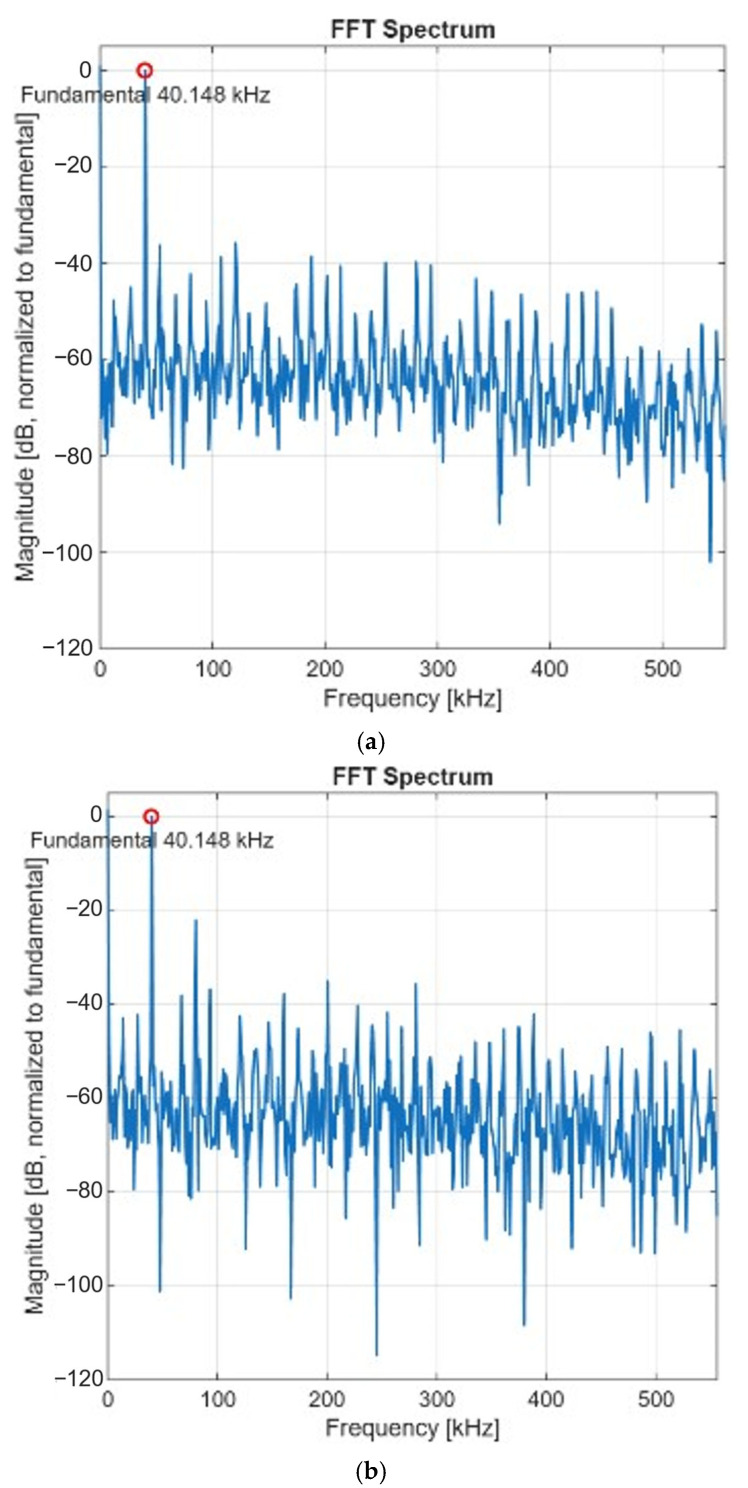
FFT spectra of (**a**) the standalone SAR ADC only, and (**b**) the complete TVC + SAR ADC system.

Figure 25 shows the FFT spectra of the standalone SAR ADC only and the proposed TVC + SAR system. The FFT spectra were normalized with respect to the fundamental tone at 40.148 kHz, where the fundamental tone was set to 0 dB. Figure 25a shows the FFT spectrum of the standalone SAR ADC, where the measured SINAD and ENOB were 26.67 dB and 4.14 bits, respectively. Figure 25b shows the FFT spectrum of the complete TVC + SAR ADC system under the same sampling condition, in which the measured SINAD and ENOB were 20.57 dB and 3.12 bits, respectively. Compared to the standalone SAR ADC, the proposed TVC + SAR ADC system exhibits increased distortion due to the nonlinearity and timing conversion error introduced by the TVC stage.

Table 1 summarizes the prior time-of-flight (ToF) sensing systems together with the proposed work. Since the prior arts target various LiDAR applications and adopt different architectures such as ADC- and TDC-based implementations, the comparison is primarily intended to provide system-level architectural overview rather than strict specification-to-specification benchmark comparison. Nonetheless, Table 1 highlights the differences in backend conversion strategy, implementation complexity, power consumption, and scalability relevant to LiDAR receiver designs.

Ref. [18] employed pulse-shaping with zero-crossing detection to achieve a wide dynamic range (~1:50,000). However, the proposed work significantly reduces analog front-end complexity by eliminating the need for LC resonators and nonlinear feedback TIAs, while maintaining a scalable architecture.

Ref. [19] achieved high timing accuracy using a multi-channel TDC with additional time-domain parameters for walk error compensation. Meanwhile, the proposed architecture avoids complex TDC calibration and multi-parameter extraction, resulting in lower implementation complexity and improved energy efficiency.

Ref. [20] adopted a hybrid sampling-based readout (SSA-TDC) to capture waveform information, whereas the proposed design eliminates the need for high-speed sampling arrays and memory, thereby reducing hardware overhead and simplifying the signal processing chain.

Ref. [21] utilized a multiplexed ADC/TDC structure to improve integration efficiency. However, the proposed work further simplifies the architecture by directly converting time information into the voltage domain, avoiding duplicated conversion paths and synchronization issues.

Overall, the proposed TVC–SAR ADC-based LiDAR receiver achieves a more favorable tradeoff among circuit complexity, power consumption, and scalability by removing the need for high-speed TDCs and complex hybrid architectures, while still enabling efficient time-interval digitization.

## 4. Conclusions

We have proposed a low-power LiDAR receiver topology based on a time-to-voltage converter (TVC) followed by a 5-bit SAR ADC, implemented in a 180 nm CMOS process. By translating the time-intervals into the voltage domain, the proposed topology eliminates the need for a complex TDC-based architecture and enables a simpler and more energy-efficient back-end conversion. The proposed topology integrates on-chip APDs, a CTLA, a time-gating circuit, a ramp generator, a PDH, and a V_CM_-based SAR ADC with a binary-weighted C-DAC and strong-arm comparator. In addition, dedicated digital logic and a reset generator were exploited to ensure the proper timing coordination and reliable operations. With its reduced architectural complexity, compact implementation, and low power consumption, the proposed TVC-SAR receiver topology provides an energy-efficient solution for time-interval detection and is well suited for low-power and area-constrained LiDAR sensor applications.

## Figures and Tables

**Table 1:** Performance comparison of the proposed TVC-SAR with the previously reported ToF systems.

Parameters	[18]	[19]	[20]	[21]	This Work
Architecture	AFE + TDC	TDC	TDC	ADC + TDC	TVC + SAR ADC
Target	Wide-DR	High precision	Multichannel	Long-range	Short-range
Time Conv.	Zero-crossing	Direct time	Sampling	VTC + TDC	TVC
Technology (nm)	350	350	180	65	180
Supply Voltage (V)	3.3	3.3	3.3	1.2/1.8	1.8
Resolution (ps)	~100	~10	~180	6.25	810
Power dissipation (mW)	155	~150	45 (per channel)	12.44	13.0
Core area (mm^2^)	N/A	10 (Whole IC)	2.52	0.06	0.148
Complexity	Very high	High	High	High	Low
Digitization	TDC	TDC	SSA-TDC	ADC/TDC	SAR ADC

## Data Availability

The data supporting this study’s findings are available from the corresponding author upon reasonable request.
